# Maxillofacial fractures and craniocerebral injuries – stress propagation from face to neurocranium in a finite element analysis

**DOI:** 10.1186/s13049-015-0117-z

**Published:** 2015-04-21

**Authors:** Heike Huempfner-Hierl, Andreas Schaller, Thomas Hierl

**Affiliations:** Department of Oral and Maxillofacial Plastic Surgery, Leipzig University, Liebigstrasse 12, 04103 Leipzig, Germany

**Keywords:** Facial fractures, Finite element analysis, Craniocerebral injuries, Stress propagation

## Abstract

**Background:**

Severe facial trauma is often associated with intracerebral injuries. So it seemed to be of interest to study stress propagation from face to neurocranium after a fistlike impact on the facial skull in a finite element analysis.

**Methods:**

A finite element model of the human skull without mandible consisting of nearly 740,000 tetrahedrons was built. Fistlike impacts on the infraorbital rim, the nasoorbitoethmoid region, and the supraorbital arch were simulated and stress propagations were depicted in a time-dependent display.

**Results:**

Finite element simulation revealed von Mises stresses beyond the yield criterion of facial bone at the site of impacts and propagation of stresses in considerable amount towards skull base in the scenario of the fistlike impact on the infraorbital rim and on the nasoorbitoethmoid region. When impact was given on the supraorbital arch stresses seemed to be absorbed.

**Conclusions:**

As patients presenting with facial fractures have a risk for craniocerebral injuries attention should be paid to this and the indication for a CT-scan should be put widely. Efforts have to be made to generate more precise finite element models for a better comprehension of craniofacial and brain injury.

**Electronic supplementary material:**

The online version of this article (doi:10.1186/s13049-015-0117-z) contains supplementary material, which is available to authorized users.

## Abstract (de)

**Hintergrund:**

Frakturen des Gesichtsschädels gehen häufig mit intrakraniellen Verletzungen einher. Deshalb erschien es interessant, die Weiterleitung und Verteilung von Spannungen, wie sie bei einem Faustschlag auftreten, vom Gesichtsschädel zum Hirnschädel in einer Finite Elemente Analyse zu untersuchen.

**Methoden:**

Ein Finite Elemente Modell des menschlichen Schädels ohne Unterkiefer, welches aus zirka 740,000 tetraedrischen Volumeneinheiten bestand, wurde entwickelt. Die Einwirkung einer Kraft, die einem Faustschlag entsprach, auf den Infraorbitalrand, die Nasoorbitoethmoidregion und den supraorbitalen Bogen wurden simuliert. Die Weiterleitung der Spannungen wurde in einem zeitlichen Verlauf dargestellt.

**Ergebnisse:**

Die Finite Elemente Simulation zeigte von Mises-Spannungen oberhalb des Wertes, ab dem Gesichtsschädelknochen frakturieren, im Bereich der Krafteinleitung und Fortleitung von Spannungen in Richtung auf die Schädelbasis in beachtlicher Höhe bei Auftreffen des Impaktors auf den Infraorbitalrand oder die Nasoorbitoethmoidregion. Bei Auftreffen der Kraft supraorbital scheinen die Spannungen absorbiert zu werden.

**Schlussfolgerungen:**

Da Patienten mit Gesichtsschädelfrakturen ein Risiko für Schädel-Hirn-Traumata aufweisen, sollte eine entsprechende Überwachung erfolgen. Die Indikation für CT-Untersuchungen sollte großzügig gestellt werden. Genauere Finite Elemente-Modelle sind zum besseren Verständnis kraniofazialer Frakturen und Hirnverletzungen notwendig.

## Background

Severe facial trauma is often associated with intracerebral injuries. McLean described inertial loading and head acceleration as a cause of brain injury [1]. It is obvious that impact on the facial skeleton results in head acceleration. Many studies report on statistical analyses concerning patterns of facial fractures and probability of intracranial haemorrhage. Bellamy et al. reported on 3,291 patients with midfacial fractures and found that 21.3% of them had intracranial injuries, 6.3% died. Here the cumulative incidence of intracranial injury of simple midface fractures was 6.3% and that of complex midface fractures was 11.9% [2,3]. In their study on 6,117 patients who were admitted for blunt trauma Plaisier et al. found that 48% of patients who died of neurologic injury showed midfacial fractures [4]. A potential mechanism is ruptures of intracranial vessels [5]. In a further large study Salentijn et al. reported on 579 trauma patients with facial fractures, 8.1% of them had also intracranial injuries [6,7].

In patients suffering from high velocity impacts like car accidents the mechanisms of brain injury seem obvious, and the patients have to undergo a cranial CT scan to detect intracranial haemorrhage. But question arises concerning impairment in patients with smaller impacts.

Several authors [5,8] report on a high percentage of intracerebral injuries in patients presenting with facial fractures without any neurological impairment and recommend a wider indication for cranial CT scan than has been previously published [9]. So it seemed to be of interest to simulate fistlike impacts on the midface and the upper face in a finite element analysis to gain information about the dispersion of stresses and to investigate the aetiopathogenesis of craniocerebral injuries after blunt facial skull trauma.

## Methods

To conduct finite element analyses corresponding with a traumatic scenario of fistlike impacts on the midface and the upper face a finite element model (FE model) of the skull without mandible was generated. The model consisted of 736,934 tetrahedral shaped 10-node elements and was based on the CT-dataset of a 34 years old white Caucasian male without any pathologies (Siemens Volume Zoom Plus, 1 mm contiguous slicing). This CT-dataset was exported into VRML data format after manual segmentation, triangulated (VWorks 4.0® Cybermed, Korea) and exported to ANSYS ICEM CFD 12.0.1® (ANSYS Inc., Canonsburg, PA, U.S.A.) [10]. One of the specific characteristics of the model was to assign individual bone material parameters to each element by transforming grey scale values of the CT Hounsfield scale into information about bone density. By using a BoneMat script Young’s moduli for each element were computated [11,12].

Regarding the impact simulating a punch of fist, a virtual brass impactor (weight of 412 grams, density of 8.4 grams per cubic centimetre, Young’s modulus of 100,000 megapascal, Poisson ratio of 0.37) was modelled in accordance with the experiments of Waterhouse et al. [13]. Impact velocity was set to six meters per second.

Because of time dependency of interaction between skull bone and impactor a transient mode of simulation was applied, which in contrast to static finite element analysis reflects the gradient impact, which seems more realistic in fast phenomena.

The model was fixed at the occipital condyles in all degrees of freedom. For the yield criterion of skull bone von Mises stresses of 150 megapascal are accepted [14].

**Figure 1:**
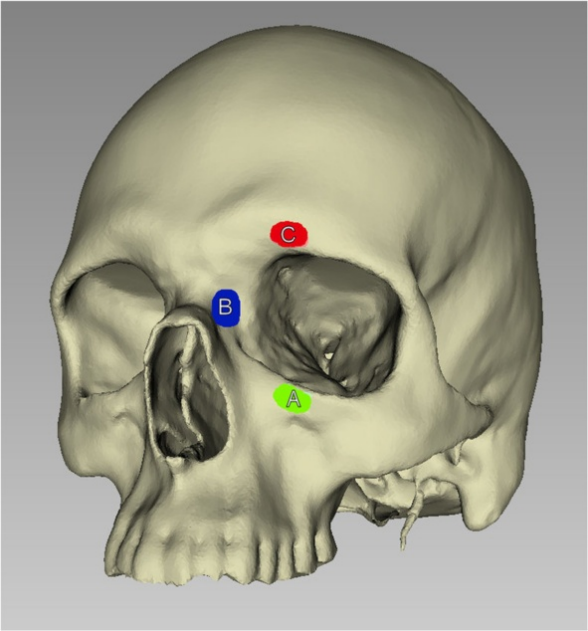
Site of impact by virtual impactors: **A)** Impact on the medial orbital rim (green); **B)** Impact on the nasoorbitoethmoid region (blue); **C)** Impact on the supraorbital arch (red).

Three different areas of impact were chosen. In the first the impactor hit the facial bone in the medial third of the infraorbital rim, in the second in the junction area between nasal bone, maxillary nasal process and lacrimal bone, in the third on the supraorbital arch (see Figure 1, A, B, C). Impact was identical concerning the three sites. Stress propagation is depicted in a time-dependent display for each scenario.

According to the regulations of our institutional review board no approval of this investigation has been necessary.

## Results

Finite element simulation revealed von Mises stresses beyond the yield criterion of facial bone, which correlates with fractures at the site of impact [15].

In this study main attention shall be paid to stress propagation towards cranium and skull base caused by an impact, which would lead to rather simple facial fractures.

### Impact on the infraorbital rim

**Figure 2:**
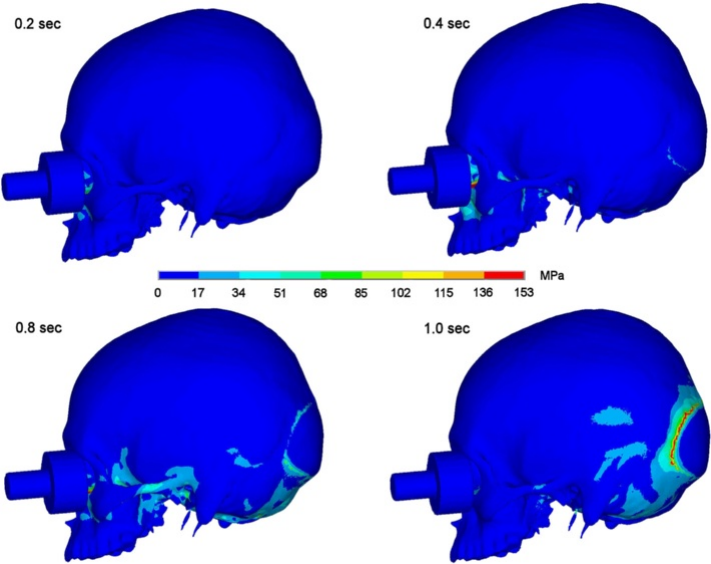
Stress propagation after impact on the medial infraorbital rim. Stress distribution 0.2, 0.4, 0.8 and 1.0 sec after impact in lateral view.

**Figure 3:**
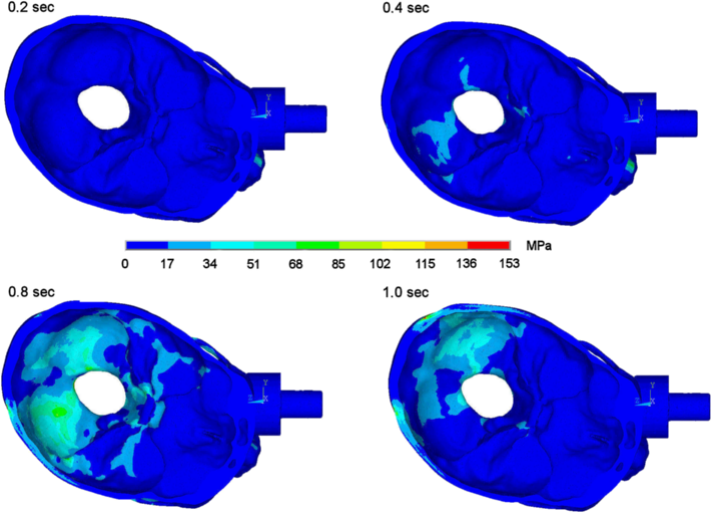
Stress propagation after impact on the medial infraorbital rim. Stress distribution 0.2, 0.4, 0.8 and 1.0 sec after impact in view onto skull base.

Time-dependent stress propagation caused by a fistlike impact on the medial third of the infraorbital rim causes first maximum stress on the site of impact. Here the threshold for bone is exceeded, which stands for bone fracture in this area. Then stresses propagate in an anterior-posterior direction over skull base with processus styloidei and zygomatic arch to the occipital bone. Considerable stress propagation is seen in the zygomatic arch and in the skull base, especially in the posterior fossa and in the clivus area. In Figures 2 and 3 the dispersion of stresses is shown 0.2, 0.4, 0.8 and 1.0 seconds after impact on the medial infraorbital rim.

### Impact on the nasoorbitoethmoid region

**Figure 4:**
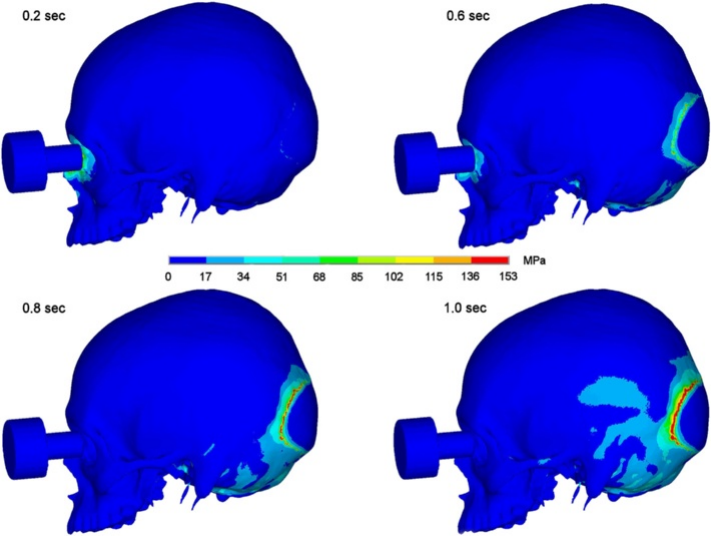
Stress propagation after impact on the nasoorbitoethmoid region. Stress distribution 0.2, 0.6, 0.8 and 1.0 sec after impact in lateral view.

**Figure 5:**
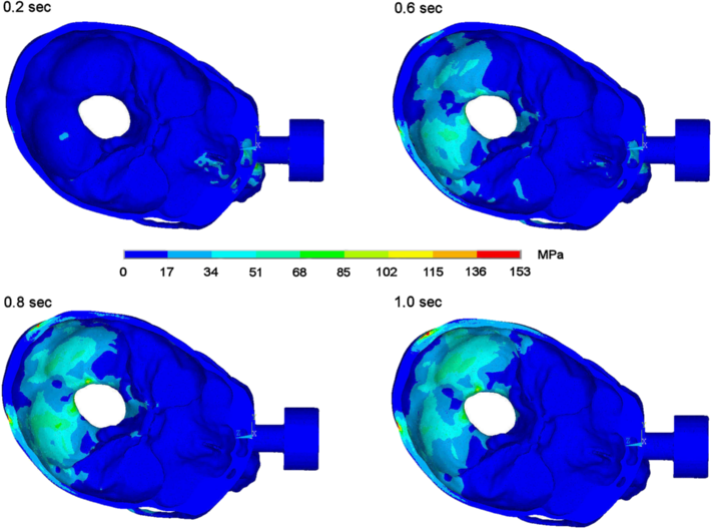
Stress propagation after impact on the nasoorbitoethmoid region. Stress distribution 0.2, 0.6, 0.8 and 1.0 sec after impact in view onto skull base.

By giving a fistlike impact onto the junction area between nasal bone, maxillary nasal process and lacrimal bone maximum stress was seen at the site of impact correlating with a fracture of this area. Moreover, stresses propagate to the midface in the Le Fort I-plane, to the pterygomaxillary junction, to skull base and occipital bone and also in direction to the zygomatic arch. Also in this scenario high stresses were seen in the occipital bone 1.0 second after impact. In Figures 4 and 5 the dispersion of stresses is shown 0.2, 0.6, 0.8 and 1.0 seconds after impact on the nasoorbitoethmoid region.

### Impact on the supraorbital arch

**Figure 6:**
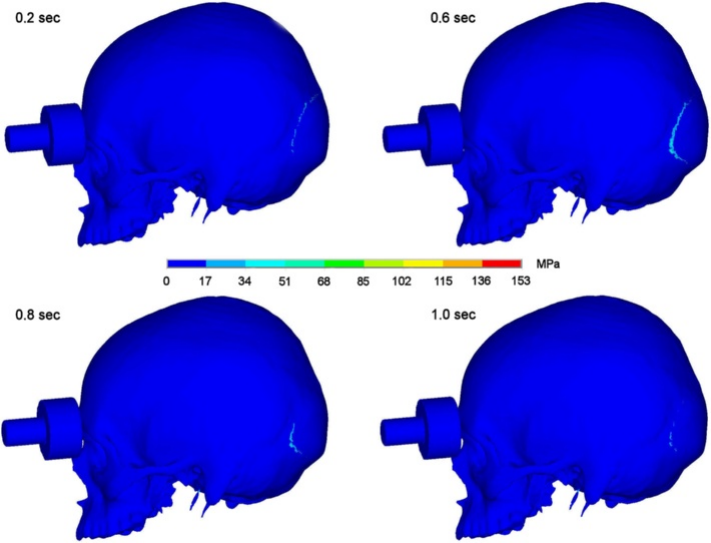
Stress propagation after impact on the supraorbital arch. Stress distribution 0.2, 0.6, 0.8 and 1.0 sec after impact in lateral view.

**Figure 7:**
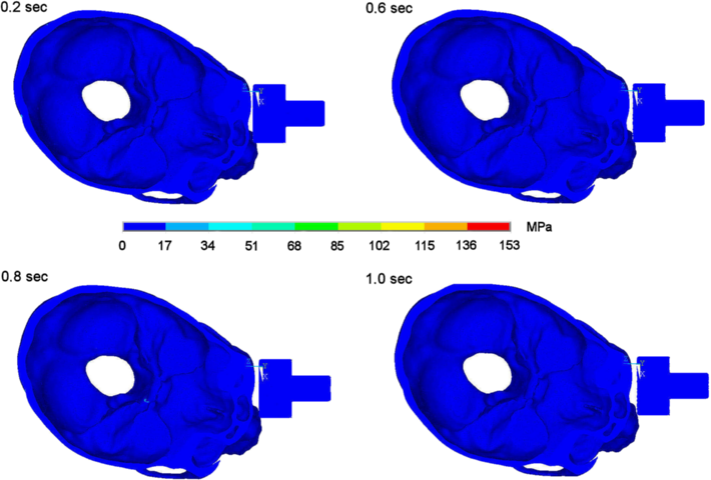
Stress propagation after impact on the supraorbital arch. Stress distribution 0.2, 0.6, 0.8 and 1.0 sec after impact in view onto skull base.

Fistlike impact on the supraorbital arch produces maximum stresses at the site of impact. Then stress propagates into the orbit in direction to the optic canal and to the occipital bone. Stresses in the occipital bone caused by impact on the supraorbital arch are much smaller than in the scenarios described before and reach about 50 megapascal 0.6 seconds after impact. That means that stresses are neutralised faster than in both other scenarios and remain on a lower level. In Figures 6 and 7 the dispersion of stresses is depicted 0.2, 0.6, 0.8 and 1.0 seconds after impact.

## Discussion

Generally it is very difficult to perform studies concerning traumatic scenarios in a realistic and valid set-up because of technical and ethical reasons. The well-known and fundamental studies published by Le Fort in 1901 [16,17] would not be practicable because of absence of cadavers for studies. Moreover, Le Fort’s study design supposably would hardly pass an institutional review board today. The validity of cadaver studies is limited, as the specimen will have undergone postmortal alterations and in most cases will be destroyed by impacting trauma so that results are not really reproducable. Experiments in laboratory rhesus monkeys concerning cerebral concussion after sagittal plane angular acceleration deliver information about the viscoelastic behaviour of bridging veins [18], but are only partially transferable to biomechanics of the human skull and impacting forces in a real trauma. The use of animal models can be discussed controversially in general.

About thirty years ago first steps were made towards finite element analysis, which was limited by computing capacity. This led to rather simple 2D-models [19], data derived from cadaver data in most finite element models [20,21].

Approaches have been made to analyse brain-skull interaction during and after trauma. Here it is highly desirable to have a very fine finite element model of the skull, where all tissues and their mechanical behaviour could be incorporated.

Unfortunately such a model does not exist by now. It is very difficult to gain valid information concerning biomechanical properties of involved tissues, which probably differ depending on age, gender and ethnicity. Even if these questions were answered and all material parameters were available, such a precise model would require an enormous computing capacity. Another important point is the origin of the data used for generating a finite element model. Still most models presented in literature derive from cadaver data. Postmortal alterations and the fact that the age of cadavers mostly is not the typical age of patients suffering from facial skull fractures is often neglected.

Zong et al. [22] presented a three-dimensional finite element model of the human head, which consisted of brain, inner and outer layer of the skull, diploë, cerebrospinal fluid and cervical elements. Even if this model might be sophisticated concerning different tissues, it consists only of a small number of elements and is rather simplified. Nevertheless, it delivers valuable information, as it showed dynamics by using a vector quantity to display power flow in magnitude and direction. Moreover they showed that a power flow exists in frontal impacts in three directions, namely to the skull base, along the cranial vault and in direction to the brain. These findings correspond well with our own findings, where stresses propagate from facial skull to viscerocranium.

A sophisticated FE model of the skull, containing scalp, outer table, spongious bone, inner table, cerebrospinal fluid and brain was presented by Hamel et al. [23] in a forensic study about skull fractures caused by falls in 2013. This model consisted of 497,000 elements and data derived from the CT-scan of a 30 years old male, which is in line with our own finite element model. Unfortunately, their study reports only about skull fracture but not about accompanying brain injuries.

In 2013 Mao et al. reported on another very detailed and high quality finite element human head, which was integrated into the Global Human Body Models [24]. This consisted of skull, brain, falx, tentorium, cerebrospinal fluid spaces and even bridging vein meshes. Data derived from the CT-data of an average American. The model had 270,552 elements in total. Even in this highly validated FE model the authors point out that mechanical characteristics of skull-brain interface structures are not fully understood by now, especially how they interact under in vivo conditions.

There is no controversy that brain damage may not only result from direct trauma to the brain tissue like in open brain or in missile injuries, but also from indirect trauma. In most cases intracranial haemorrhage will be found causative for brain damage. According to pathological data published by Crooks [25] extradural haematomas count for five to fifteen percent, subdural haematomas for 26 to 63% and intracerebral haematomas for fifteen percent in severe head injuries. The most prevalent haemorrhage derives from tearing of bridging veins. Supposably similar rates will apply to non-pathological cases.

A threshold for rupture of vessels cannot really be defined, but it is known that the risk of vessel rupture increases with angular acceleration. Concerning time factor it could be shown that an acceleration pulse greater than 5 msec would also result in failure of the visco-elastic behaviour of bridging veins [18,25].

Another reason for brain damage could be axonal injury. Unfortunately it is even more difficult to characterize a threshold for this. Attempts have been made in using small [26] and large animal models. Miller et al. analyzed the relationship between lesion patterns as a sign for diffuse axonal injury and loading conditions in a minipig model [27], but here the pigs underwent repeated tangential acceleration. It seems doubtful that such a study design really correlates with direction and extent of forces applied to human beings in assaults or even car accidents. Bain et al. tried to characterize thresholds for traumatic axonal damage in a guinea pig model [28]. They analyzed the dynamic optic nerve elongation and concluded that axonal thresholds deriving from this analysis can be directly applied to human head injury. Obviously it is a noble goal to gain threshold properties for several tissues to have the possibility to integrate them into FE models, but we have certain doubts, whether tearing on a guinea pig’s eye will deliver the desired information. So one always will get to the basic problem that it is extremely difficult to simulate traumatic scenarios in a valid and reproducable manner.

The finite element model presented in this study derived from the CT-data of a 34 years old man and consisted of nearly 740,000 tetrahedrons. This represents a very high resolution. As individual bone parameters according to grey scale values had been attributed and a transient mode of simulation had been chosen, this resulted in a model, which is supposed to give valid and reliable information concerning stress propagation in the human skull.

Our results show stress propagation from facial skull towards skull base in impacts that would cause fractures of the infraorbital rim, the orbital floor and in the nasoorbitoethmoid region. That corresponds to types of fractures which are frequently encountered in maxillofacial surgery. Stresses reach about 150 megapascals in the occipital bone and about 100 megapascals in the skull base.

150 megapascals are seen as a threshold for facial bone to fail and fracture [14]. As occipital bone is by far thicker than facial bone and forces required for fractures of the occipital bone are tenfold to forces required for fractures of facial bone [29], this does not mean that bone will also fracture in the occiput, but it is a strong hint that there are considerable stresses, which might cause brain damage and laceration or disrupture of bridging veins. Stresses of 150 megapascals correspond to a typical single fisticuff. A further interpretation of these results is difficult. Brain injuries are expected, when the peak brain pressure is higher than 173 kPa [30]. But there is no possibility to conclude from stress in bone to brain pressure directly, as little is known about the mechanical behaviour of skull brain interface. Less is known about the clinically relevant behaviour of bridging veins. Even in more recent studies, which use highly developed FE models with simulation of all tissues, no conclusions about propagation of stresses from bone to bridging vessels and brain are possible [31]. Moreover most studies deal with direct impact to the skull and the brain beneath, whereas the subject of our study focusses on stresses propagated from facial impact to skull base. Further studies to deal with these questions are required.

Concerning impacts to the supraorbital arch we have seen that there is nearly no propagation of stresses to the skull base in comparison to the infraorbital rim and the nasoorbitoethmoid region, although impact was identical and produced fractures at the side of impact in all three scenarios. Stresses seem to be absorbed in the supraorbital region. So the supraorbital arch is a structure, which is able to carry loads from impacts and to protect skull and brain. This phenomenon has been reported in an earlier investigation [32].

The FE model used in this study has its limitations, as it is a model consisting only of midfacial and skull bone, but not of the brain and other head tissues. Nevertheless, it delivers valid information about stress propagation within the skull. Its informative value is supported by many studies about prevalence of craniocerbral injuries and even deaths in patients with facial fractures. Thorén et al. reported on associated injuries in patients with facial factures and found that a quarter of these patients had associated injuries, of which 11% were brain injuries [33]. Kaiser published a case report on death in an assault victim presenting with a fracture of the orbital wall and lacerations of the chin because of extensive basal subarachnoidal bleeding [34].

There is discussion whether the midface has the function of a cushion and might absorb forces to the facial skull to protect the brain, or whether forces will propagate in direction to skull base and brain [35,36]. Keenan et al. presented a case–control study of 3849 injured bicyclists and five scene deaths and found an odds ratio of 9.9 for the risk of intracranial injury in association with facial fractures. They interpreted facial fractures as signs for increased risk of brain injury [36]. This is in accordance with our own findings concerning stress propagation.

Adamec et al. concluded in their study about the injury risk of a headbutt that a headbutt, which might be comparable to a fisticuff, will unlikely cause lethal injuries, but they also point out that under certain cirumstances, e.g. support of the victim’s head when standing against a wall or lying on the floor, life-threatening injuries could occur [29]. As they used volunteers, who were obliged to perform a headbutt, for their biomechanical studies in our opinion occurring forces might have been smaller than in real assault situations. According to our own finite element studies and also according to clinical experience headbutts and fisticuffs definitely may lead to fractures and brain contusion. Salentijn et al. also saw a clear association of facial trauma with traumatic brain injury [6,7].

## Conclusions

According to literature and our presented results concerning stress propagation, there is a certain risk of incidence of craniocerebral injuries even in patients presenting only with minor fractures of the facial skull.

Impacts on the supraorbital arch encounter a construction type which seems to be suited for the absorption of blows, whereas blunt injuries to the infraorbital rim and the nasoorbitoethmoid region bear a higher risk for craniocerebral injuries by dispersing stresses. So in patients presenting with fractures in these areas high attention should be paid to craniocerebral injuries and there should be a wide indication for CT scans to prevent to miss severe brain damage especially in cases with a time delay.

Futher efforts have to be made to create more precise finite element models incorporating all necessary tissues for better understanding of craniofacial and brain injury.
